# *SMAD3* rs17228212 Gene Polymorphism Is Associated with Reduced Risk to Cerebrovascular Accidents and Subclinical Atherosclerosis in Anti-CCP Negative Spanish Rheumatoid Arthritis Patients

**DOI:** 10.1371/journal.pone.0077695

**Published:** 2013-10-21

**Authors:** Mercedes García-Bermúdez, Raquel López-Mejías, Fernanda Genre, Santos Castañeda, Carlos González-Juanatey, Javier Llorca, Alfonso Corrales, José A. Miranda-Filloy, Javier Rueda-Gotor, Carmen Gómez-Vaquero, Luis Rodríguez-Rodríguez, Benjamín Fernández-Gutiérrez, Dora Pascual-Salcedo, Alejandro Balsa, Francisco J. López-Longo, Patricia Carreira, Ricardo Blanco, Isidoro González-Álvaro, Javier Martín, Miguel A. González-Gay

**Affiliations:** 1 Instituto de Parasitología y Biomedicina López-Neyra, IPBLN-CSIC, Granada, Spain; 2 Epidemiology, Genetics and Atherosclerosis Research Group on Systemic Inflammatory Diseases, Rheumatology Division, Hospital Universitario Marqués de Valdecilla, IFIMAV, Santander, Spain; 3 Department of Rheumatology, Hospital Universitario la Princesa, IIS-Princesa, Madrid, Spain; 4 Cardiology Division, Hospital Lucus Augusti, Lugo, Spain; 5 Department of Epidemiology and Computational Biology, School of Medicine, University of Cantabria, and CIBER Epidemiología y Salud Pública (CIBERESP), IFIMAV, Santander, Spain; 6 Division of Rheumatology, Hospital Xeral-Calde, Lugo, Spain; 7 Department of Rheumatology, Hospital Universitario Bellvitge, Barcelona, Spain; 8 Department of Rheumatology, Hospital Clínico San Carlos, Madrid, Spain; 9 Department of Rheumatology, Hospital Universitario la Paz, Madrid, Spain; 10 Department of Rheumatology, Hospital Universitario Gregorio, Marañón, Madrid, Spain; 11 Department of Rheumatology, Hospital Universitario, Madrid, Spain; University of Michigan, United States of America

## Abstract

Rheumatoid arthritis (RA) is a complex polygenic inflammatory disease associated with accelerated atherosclerosis and increased risk of cardiovascular (CV) disease. Previous genome-wide association studies have described *SMAD3* rs17228212 polymorphism as an important signal associated with CV events. The aim of the present study was to evaluate for the first time the relationship between this gene polymorphism and the susceptibility to CV manifestations and its potential association with the presence of subclinical atherosclerosis assessed by the evaluation of carotid intima-media thickness (cIMT) in patients with RA.

**Methods:**

One thousand eight hundred and ninety-seven patients fulfilling classification criteria for RA were genotyped for *SMAD3* rs17228212 gene polymorphism through TaqMan genotyping assay. Also, subclinical atherosclerosis determined by the assessment of cIMT was analyzed in a subgroup of these patients by carotid ultrasonography.

**Results:**

No statistically significant differences were observed when allele frequencies of RA patients with or without CV events were compared. Nevertheless, when RA patients were stratified according to anti-cyclic citrullinated peptide (anti-CCP) status, we found that in RA patients who were negative for anti-CCP antibodies, the presence of C allele of *SMAD3* rs17228212 polymorphism conferred a protective effect against the risk of cerebrovascular accident (CVA) after adjustment for demographic and classic CV risk factors (HR [95%CI]=0.36 [0.14–0.94], *p*=0.038) in a Cox regression model. Additionally, correlation between the presence of C allele of *SMAD3* rs17228212 polymorphism and lower values of cIMT was found after adjustment for demographic and classic CV risk factors (*p-value*=0.0094) in the anti-CCP negative RA patients.

**Conclusions:**

Our results revealed that *SMAD3* rs17228212 gene variant is associated with lower risk of CVA and less severe subclinical atherosclerosis in RA patients negative for anti-CCP antibodies. These findings may have importance to establish predictive models of CV disease in RA patients according to anti-CCP status.

## Introduction

Rheumatoid arthritis (RA) is a complex autoimmune disease associated with progressive disability, systemic complications and early death. Mortality is higher among RA patients than in the general population, and cardiovascular (CV) complications remain a major challenge [1]. Atherosclerosis is the main cause of increased CV morbidity and mortality in RA patients. As well as traditional CV risk factors, chronic systemic inflammation plays a pivotal role in the development of accelerated atherosclerosis observed in RA [2]. Moreover, recent studies have also highlighted the implication of genetic factors in the susceptibility to and/or risk of accelerated atherosclerosis of patients with RA [3-5].

Genome-wide Association studies of coronary artery disease (CAD) performed in Caucasian populations have identified a number of genetic variants that were associated with this pathology. In this regard, variant rs17228212 of *SMAD3* located in 15q22.33 chromosomal region was detected after a combined meta-analysis between the Wellcome Trust Case Control Consortium study and The German Myocardial Infarction Family Study, with high probability of a true association [6]. *SMAD3* gene encodes an intracellular signal transducer and transcriptional modulator activated by transforming growth factor-beta (TGF-β) and activin type 1 receptor kinases. Smad3 is directly phosphorylated by the activated type I receptors on its C-terminal Ser-Ser-X-Ser motif. This C-terminal phosphorylation allows binding to common mediator Smads and translocation to the nucleus where they can recruit transcriptional co-activators or co-repressors and regulate TGF-β target genes [7]. In the immune system, TGF-β modulates the balance of proinflammatory and anti-inflammatory T-cells through a complex set of interactions. SMAD3 has an essential role in downregulating T-cells and increasing expression of FoxP3, an essential step in the differentiation of regulatory T-cells [8]. Imbalance of proinflammatory Th17 and regulatory T-cells has been reported in acute coronary syndrome [9].

Besides, a *SMAD3* haplotype has been associated with Kawasaki disease, a systemic vasculitis disease associated with cardiovascular sequelae [10]. Moreover, gene variants in *SMAD3* have been associated with inflammatory bowel disease and asthma [11].

Taking into account all these considerations, in the present study we aimed to assess, for the first time, the potential implication of the *SMAD3* rs17228212 polymorphism in the susceptibility to CV manifestations and its possible association with the presence of subclinical atherosclerosis assessed by the evaluation of carotid intima-media thickness (cIMT) using carotid ultrasonography (US) in RA in a large and well characterized cohort of Spanish RA patients.

## Materials and Methods

### Patients and Study Protocol

#### Ethics Statement

A subject’s written consent was obtained according to the declaration of Helsinki, and purpose of the work was approved by the Ethics Committee of Galicia (Spain). The Ethics Committees of the Hospital Universitario Marqués de Valdecilla (Santander), Hospital Universitario Bellvitge (Barcelona), Hospital Universitario La Paz, Hospital de La Princesa, Hospital Clínico San Carlos, Hospital 12 de Octubre and Hospital Universitario Gregorio Marañón (Madrid) also approved the study.

A cohort of 1897 RA Spanish patients were included in the present study. Blood samples were obtained from patients recruited from diverse Spanish Hospitals mentioned above. All the patients fulfilled the 1987 American College of Rheumatology (ACR) criteria for the classification of RA [12] and also the 2010 classification criteria for RA [13]. Between December 2009 and January 2013 patient’s clinical records were examined until patient’s death, loss of follow-up or December 1^st^, 2012.

Information on the main demographical data, clinical characteristics of the patients enrolled in the study and risk factors of patients are shown in Table **1**. Three hundred and sixty-nine (19.45%) of these 1897 RA patients had experienced clinically evident CV events: a CV event was considered to be present if the patient had ischemic heart disease (IHD) (9.76%), cerebrovascular accidents (CVA) (5.43%), heart failure (HF) (6.07%) or peripheral arteriopathy (2.53%). Clinical definitions for CV events and classic CV risk factors were described elsewhere [14,15]. Briefly, definition of IHD included acute coronary syndromes with or without persistent ST-segment elevation and chronic coronary heart disease. Data regarding the clinical presentation of HF were collected for all patients, based on the Framingham criteria [16]. A patient was considered to have a CVA when he/she had a stroke and/or transient ischemic attacks (TIAs). Strokes were classified according to their clinical features and they were confirmed by computed tomography and/or magnetic resonance imaging. TIAs were diagnosed if the symptoms were self-limited in less than 24 hours, without residual neurological damage. Peripheral arterial disease was considered present if it was confirmed by Doppler and/or arteriography [15]. Information on the clinical characteristics and risk factors of RA patients who suffered CVA are shown in Table **S1** in File S1.

**Table 1:** Demographic characteristics of the RA patients included in the study.

**Clinical Feature**	**% (n/N)**	**Absence of CV events, % (n)**	**Presence of CV events, % (n)**	***p***
Main Characteristics				
Women	74.06 (1405/1897)	77.62 (1186)	59.35 (219)	<10^-7^
Age of patients at the time of disease diagnosis, years, median (IQR)	54 (43-64)	52 (42-62)	62 (53-70)	<10^-3^
Time follow-up, years, median (IQR)	9.9 (5-16.5)	9 (4-15)	12 (6-19)	0.01
anti-CCP positive	58.71 (927/1579)	59.40 (774)	55.43 (153)	N.S.
Rheumatoid Factor positive	66.78 (1194/1788)	67.19 (981)	64.94 (213)	N.S.
Erosions	54.93 (763/1389)	55.08 (629)	54.25 (134)	N.S.
Extra-articular Manifestations	32.05 (449/1401)	31.11 (354)	36.12 (95)	N.S.
Cardiovascular Risk Factors				
Hypertension	38.49 (722/1876)	33.16 (503)	61.00 (219)	<10^-7^
Diabetes mellitus	12.72 (238/1871)	10.18 (154)	23.46 (84)	<10^-7^
Dyslipidemia	36.33 (675/1858)	33.27 (500)	49.30 (175)	<10^-7^
Obesity	20.26 (343/1693)	19.31 (268)	24.59 (75)	0.038
Smoking habit*	33.33 (619/1821)	31.85 (467)	42.82 (152)	<10^-4^

Except where indicated otherwise, values are % (n/N).

CV: Cardiovascular. IQR: Interquartile Range. Anti-CCP: anti-cyclic citrullinated peptide antibodies. N.S.: Non significant.Smoking habit*: including active smoker patients´ and patients who had ever smoked.

### Genotyping

DNA from patients was obtained from peripheral blood using standard methods. DNA patient was assessed for rs17228212 *SMAD3* polymorphism through TaqMan single nucleotide polymorphism (SNP) genotyping assay (C__33991364_10) in a 7900 HT real-time Polymerase Chain Reaction system, according to the manufacturer´ recommended conditions (Applied Biosystems, Foster City, CA, USA). Negative controls and duplicate samples were included to check genotyping accuracy.

### Carotid ultrasonography (US) examination

Measurement of the cIMT was performed in 444 patients from Lugo and Santander by carotid US. Patients from Santander were assessed using a commercially available scanner, Mylab 70, Esaote (Genoa, Italy) equipped with 7–12 MHz linear transducer and the automated software guided technique radiofrequency—Quality Intima Media Thickness in real-time (QIMT, Esaote, Maastricht, Holland)—was used [17]. Patients from Lugo were evaluated using high-resolution B-mode ultrasound, Hewlett Packard SONOS 5500, with a 10-MHz linear transducer as previously reported [18]. cIMT was measured at the far wall of the right and left common carotid arteries, 10 mm from the carotid bifurcation, over the proximal 15 mm-long segment. cIMT was determined as the average of three measurements in each common carotid artery. The final cIMT was the largest average cIMT (left or right). Agreement between the two US methods in patients with RA was high [19]. Two experts with high experience and close collaboration in the assessment of cIMT in RA from Santander (AC) and Lugo (CGJ) performed the studies.

### Statistical analysis

All genotype data were checked for deviation from Hardy-Weinberg equilibrium (HWE) using http://ihg.gsf.de/cgi-bin/hw/hwa1.pl. Both allelic and genotypic frequencies were calculated and compared by χ^2^ or Fisher tests using the StatsDirect software V2.6.6 (StatsDirect http://www.statsdirect.com, England: StatsDirect 2008). Strength of associations between CV events and alleles were estimated using Odds Ratios (OR) and 95% Confidence Intervals (CI), via multiple logistic regression; estimates were further adjusted for sex, age at RA diagnosis, time of follow-up and classic CV risk factors (hypertension, diabetes mellitus, dyslipidemia, obesity, and smoking habit).

The relationship between *SMAD3* rs17228212 genotypes and CV events which appeared in the follow-up was tested using Cox proportional hazards model, adjusting for sex, age at RA diagnosis and traditional CV risk factors (hypertension, diabetes mellitus, dyslipidemia, obesity, and smoking habit). For that purpose, we used CC genotype as reference; the end of follow-up was the first date among the end of the study (December-2012), date of death, or date of CV event. Follow-up time was estimated as the difference between the RA diagnosis date and the end of follow-up. Patients without CV event in the follow-up time or dying by any other causes different from CV events were considered as censored. Results are expressed as Hazard Ratios (HR) with 95% Confidence Interval (CI). Survival curves were constructed using the Kaplan–Meier method.

The association between genotypes of the *SMAD3* rs17228212 gene variant and cIMT was tested using unpaired T-test to compare between 2 groups, and one-way analysis of variance (ANOVA) to compare among more than two groups. Moreover, we also assessed the association between cIMT and alleles using analysis of covariance (ANCOVA) adjusting for sex, age and duration of the disease at the time of the carotid US study and traditional CV risk factors.

Statistical significance was defined as p<0.05. All analyses were performed with STATA statistical software 12/SE. (Stata Corp., College Station, TX, USA).

## Results

Genotyping success rate in our study was higher than 96.5%. No deviation from Hardy-Weinberg equilibrium (HWE) was detected (*p*=0.23).

As shown in Table **1**, patients with CV events were older at the time of disease diagnosis, were more likely to be men, and more often had hypertension, diabetes mellitus, dyslipidemia and obesity. In addition, smoking habit (defined as those patients who smoked at the time of disease diagnosis, during the follow-up or who had smoked within 10 years before the onset of RA symptoms or disease diagnosis) was more frequent in men than in women RA patients (58.88% *vs.* 25.99%).

No differences were observed when allele frequencies from patients with or without CV events were compared (MAF: 0.269 and 0.262, respectively; *p*=0.70). Results from an adjusted logistic regression model did not show statistically significant association: OR (95%CI)=0.88 (0.68-1.13), *p*=0.31. Neither a statistically significant result was obtained applying a Cox regression model to estimate the influence of the *SMAD3* rs17228212 genetic variant on risk of CV disease, which was adjusted for sex, age at the time of RA diagnosis, and traditional CV risk factors as potential confounders: HR [95%CI]=0.93 [0.74-1.18], *p*=0.56. Neither association was detected for the risk of developing IHD (*p*=0.21), CVA (*p*=0.30), HF (*p*=0.27), or peripheral vascular events (*p*=0.27) in the whole RA patient´ cohort.

Nevertheless, when the cohort of RA patients was stratified according to presence or absence of anti-cyclic citrullinated peptide (anti-CCP) antibodies, differences in the risk of CV events in general (as a whole) or in the risk of suffering any of the different subtypes of CV events (IHD, CVA or HF) in patients who were negative for these antibodies were observed (Table **2**). In a further step, we performed a Cox regression model to account for the variation of risk of the first CV event through time according to allele C of rs17228212 *SMAD3* variant: in the subgroup of anti-CCP negative RA patients the presence of C allele conferred a protective effect against CVA after result adjustment for sex, age at the time of RA diagnosis, and traditional CV risk factors (HR [95%CI]=0.36 [0.14–0.94], *p*=0.038), while C allele carriers who were positive for anti-CCP antibodies did not show any reduction in the risk of CVA (HR [95%CI]=1.08 [0.55–2.10], *p*=0.82) (Table **3**). Similar results were found when Kaplan-Meier survival curves were constructed (**Suppl. Figures A-F** in File S1). No other significant results were obtained in the Cox model for any of the other CV events subtypes (data not shown).

**Table 2:** Differences in the minor allele frequencies of *SMAD3* rs17228212 polymorphism between RA patients with or without CV events, Ischemic Heart Disease (IHD), Cerebrovascular Accident (CVA) or Heart Failure (HF).

			CV event				IHD			CVA					HF	
		MAF (%)	*p*	p*		MAF (%)	*p*	p*	MAF (%)		*p*	p*	MAF (%)	*p*		p*
Anti-CCP Negative	absence	27.36				26.97			27.33				26.91			
	presence	25.42	0.54	0.035		27.19	0.96	0.25	21.79		0.29	0.011	29.49	0.62		0.93
Anti-CCP Positive	absence	25.74				26.40			26.30				25.92			
	presence	29.45	0.19	0.97		25.00	0.72	0.27	28.05		0.73	0.81	32.00	0.18		0.97

* p: *p*-value in the logistic regression model adjusted for sex, age at RA diagnosis, follow-up time and traditional CV risk factors (hypertension, diabetes mellitus, dyslipidemia, obesity, and smoking habit). CV: Cardiovascular; IHD: Ischemic Heart Disease; CVA: Cerebrovascular accident; HF: Heart Failure.

**Table 3:** Cox regression model to account for the risk of cerebrovascular accident (CVA).

	**CVA**	**CVA in anti-CCP positive patients**	**CVA in anti-CCP negative patients**
**HR [95%CI]; *p***	0.78 [0.49-1.24]; *p*=0.30	1.08 [0.55–2.10]; *p*=0.82	0.36 [0.14–0.94]; *p*=0.038

HR [95%CI]: Hazard Ratios with 95% Confidence Interval. p: p-value. Anti-CCP: anti-cyclic citrullinated peptide antibodies.

Cox regression model adjusted for sex, age at RA diagnosis and traditional CV risk factors (hypertension, diabetes mellitus, dyslipidemia, obesity and smoking habit).

Finally, as shown in Table **2**, the risk of IHD or HF was increased in RA patients carrying the C allele. Although results did not reach statistical significance, this increase of C allele frequency is in line with previous results that reported an increased risk of CAD associated to C allele in individuals without RA [6].

Furthermore, analysis of the cIMT in anti-CCP negative patients showed slight differences in this parameter as exposed in Table **4**. Regarding this, when association between *SMAD3* rs17228212 polymorphism and cIMT values was adjusted for potential confounders in the ANCOVA model, results disclosed that the presence of the C allele in the subgroup of anti-CCP negative RA patients was associated with significantly lower cIMT values than those found in anti-CCP negative patients carrying the T allele (*p-value*=0.0094). In our model, we took into account traditional CV Framingham risk factors criteria as confounder factors. Nevertheless if we had only adjusted our model by smoking habit and hypertension and not for other traditional CV risk factors, as other authors have been recently published [20], the results still would been statistically significant for the cIMT ANCOVA model: *p*=0.014.

**Table 4:** Association between *SMAD3* rs17228212 alleles and carotid intima-media thickness according to anti-CCP status in RA patients.

	**Anti-CCP negative patients**			**Anti-CCP positive patients**		
**rs1728212 alleles**	cIMT mm, mean ± SD	*p*	p*	cIMT mm, mean ± SD	*p*	p*
T	0.71 ± 0.15 (n=262)			0.74 ± 0.17 (n=302)		
C	0.69 ± 0.16 (n=90)	0.28	0.0094	0.75 ± 0.20 (n=106)	0.80	0.33

cIMT: carotid intima-media thickness. SD: Standard deviation.

* p: *p*-value in the ANCOVA model adjusted for sex, age and duration of the disease at the time of the ultrasonography study and traditional CV risk factors (hypertension, diabetes mellitus, dyslipidemia, obesity and smoking habit).

In RA patients positive for anti-CCP antibodies, results for cIMT in the ANCOVA model were not statistically significant (Table **4**).

## Discussion

Our results indicate a potential association of the *SMAD3* rs17228212 gene variant with CVA and subclinical atherosclerosis in RA patients who are negative for anti-CCP antibodies. CVA is one of the leading causes of death and disability worldwide. cIMT is a good surrogate marker of atherosclerosis and an independent predictor of myocardial infarction and stroke both in general population [21], as well as CV events in RA patients [14].

Previous studies have shown that cIMT correlates well with pathologically and clinically defined atherosclerosis, and it is regulated by genetic factors with a heritability ranging from 30% to 60% [22,23]. Subclinical atherosclerosis has been observed in patients with RA, even in those without classic CV risk factors [14]. In this regard, several validated noninvasive imaging techniques are currently available to determine subclinical atherosclerosis in RA and to study the relation of surrogate markers with the development of atherosclerosis in patients with RA [24]. Among them, carotid US has become an affordable efficient technique to measure the presence of subclinical atherosclerosis by the assessment of cIMT. Interestingly, abnormally high values of cIMT (greater than 0.90 mm) have been found to predict the development of CV events in patients with RA after 5 years of follow-up [14].

Similarities between atherosclerosis and RA, as both are chronic inflammatory diseases that exhibit similar pathophysiological mechanisms [25,26], made conceivable the search for a potential association of *SMAD3* rs17228212 gene variant with CV disease in RA. Our results in Spanish RA population show for the first time a potential protective effect of C allele of the *SMAD3* rs17228212 polymorphism against risk of developing CVA in anti-CCP negative patients. The putative protective effect of the *SMAD3* polymorphism in anti-CCP negative patients seems to be strengthened as there is lower overall risk of CV events in anti-CCP negative patients. Additionally, a correlation between presence of C allele of rs17228212 *SMAD3* gene variant in patients who were negative for anti-CCP antibodies and lower cIMT values were found after adjustment for demographic and classic CV risk factors.

Anti-CCP antibodies are highly specific markers of RA and are detected in 58–82% of patients. In RA patients, these autoantibodies are associated with greater inflammatory activity, poorer radiologic outcome, higher frequency of extra-articular manifestations, and poorer outcomes in early arthritis [27]. Although the clinical presentation at disease initiation is very similar between patients with anti-CCP-positive and anti-CCP-negative RA, disease course, possibly disease pathogenesis [28], and genetic susceptibility [29,30] are different. Association of RA with the shared epitope is different in the two serotype subsets [29]; lately it seems that non-HLA SNPs associated with RA susceptibility are only partially shared between anti-CCP-positive and anti-CCP-negative RA patients [28], confirming the hypothesis that anti-CCP-positive and anti-CCP-negative RA are two genetically different diseases [31]. Although heritability estimates remain similar in both serological strata, the contribution of the *HLA-DRB1* shared epitope alleles differs markedly, explaining 18% and 2.4% of RA heritability in anti-CCP-positive and anti-CCP-negative patients, respectively [32]. Previous studies have shown that anti-CCP antibodies are independently associated with the development of IHD, and the risk of IHD is irrespective of the titers of anti-CCP antibodies [27]. This association may be attributed to chronic inflammation that stimulates the production of anti-CCP antibodies. Patients with RA treated with disease-modifying anti-rheumatic drugs have a lower annual incidence of acute myocardial infarction because controlling inflammation reduces vascular damage. Our results would be in agreement with previous findings as they show a decreased risk of CV complications in the anti-CCP negative RA patients, which have less radiologic severity and better clinical outcome (in our study erosions and extra-articular manifestations were present in 63.01% and 38.48%, respectively, of anti-CCP positive patients; in anti-CCP negative patients 38.00% of them have erosions and 27.57% extra-articular manifestations). It is possible that the lack of correlation between anti-CCP positive and risk of CV events could be due to the more aggressive treatment to control the disease in these anti-CCP positive patients.

Intriguingly, the association observed among anti-CCP negative RA patients in the present study is in the opposite direction of that reported by Samani et al [6]. In that study, the C allele of rs17228212 was found to confer increased risk of CAD (OR [95%CI]=1.19 [1.09-1.30]), while the present study reports a protective effect of the C allele against CVA in anti-CCP negative RA patients (HR [95%CI]= 0.36 [0.14-0.96]). Because of that, more research is necessary to address that disparity, as both entities share a common physiological base, and in both the general population and in patients with RA cIMT has shown to predict the development of CV events [14]. However, treatments in use in patients suffering those conditions are different. Therefore, a thorough treatment of this difference is warranted.

In summary, our results revealed that *SMAD3* rs17228212 gene variant is associated with lower risk of CVA and less severe subclinical atherosclerosis in RA patients negative for anti-CCP antibodies. These findings may have importance to establish predictive models of CV disease in RA patients according to anti-CCP status.

## Supporting Information

File S1 **Supporting files**.
Table S1, Demographic characteristics of the RA patients included in the study according to presence of cerebrovascular accidents. Supplementary figures, Adjusted Kaplan-Meier curves for CV events and cerebrovascular accidents.
(DOC)
