# Correction: *SMAD3* rs17228212 Gene Polymorphism Is Associated with Reduced Risk to Cerebrovascular Accidents and Subclinical Atherosclerosis in Anti-CCP Negative Spanish Rheumatoid Arthritis Patients

**DOI:** 10.1371/annotation/3fc037c1-2585-4d2b-a781-e04ab7b3bfeb

**Published:** 2013-11-07

**Authors:** Mercedes García-Bermúdez, Raquel López-Mejías, Fernanda Genre, Santos Castañeda, Carlos González-Juanatey, Javier Llorca, Alfonso Corrales, José A. Miranda-Filloy, Javier Rueda-Gotor, Carmen Gómez-Vaquero, Luis Rodríguez-Rodríguez, Benjamín Fernández-Gutiérrez, Dora Pascual-Salcedo, Alejandro Balsa, Francisco J. López-Longo, Patricia Carreira, Ricardo Blanco, Isidoro González-Álvaro, Javier Martín, Miguel A. González-Gay

Affiliation number 10 for the 15th author is incorrect. The correct affiliation is: Department of Rheumatology, Hospital Universitario Gregorio Marañón, Madrid, Spain.

In addition, affiliation number 11 for the 16th author is incorrect. The correct affiliation is: Department of Rheumatology, Hospital Universitario 12 de Octubre, Madrid, Spain. 

