# Supplementary material for: SMAD3 rs17228212 Gene Polymorphism Is Associated with Reduced Risk to Cerebrovascular Accidents and Subclinical Atherosclerosis in Anti-CCP Negative Spanish Rheumatoid Arthritis Patients
Source: PLoS One. 2013 Oct 21;8(10):e77695. doi: 10.1371/journal.pone.0077695 (PMC3804609; doi:10.1371/journal.pone.0077695)
Supplement: File S1 — Supporting files. Table S1, Demographic characteristics of the RA patients included in the study according to presence of cerebrovascular accidents. Supplementary figures, Adjusted Kaplan-Meier curves for CV events and cerebrovascular accidents. (DOC) [file pone.0077695.s001.doc]

**Suppl. Table S1.** Demographic characteristics of the RA patients included in the study according to presence of cerebrovascular accidents (CVA).

| **Clinical Features** | **Absence of CVA** | **Presence of CVA** |
| --- | --- | --- |
| Main Characteristics |  |  |
| Women | 74.48 (1337) | 66.02 (68) |
| Age of patients at the time of disease diagnosis, years, median (IQR) | 52 (42-62) | 62 (53-70) |
| Time follow up, years, median (IQR) | 9 (4-15) | 12 (6-19) |
| anti-CCP positive | 59.17 (887) | 50.62 (41) |
| Rheumatoid Factor positive | 66.65 (1129) | 69.47 (66) |
| Erosions | 54.71 (720) | 58.90 (43) |
| Extra-articular Manifestations | 31.57 (418) | 39.74 (31) |
| Cardiovascular Risk Factors |  |  |
| Hypertension | 37.07 (658) | 63.37 (64) |
| Diabetes mellitus | 12.03 (213) | 25.00 (25) |
| Dyslipidemia | 35.97 (632) | 42.57 (43) |
| Obesity | 20.22 (325) | 20.93 (18) |
| Smoking habit* | 33.53 (577) | 42.00 (42) |

Except where indicated otherwise, values are % (n). IQR: Interquartile Range.

CVA: Cerebrovascular accidents. Anti-CCP: anti-cyclic citrullinated peptide antibodies.

Smoking habit*: including active smoker patients and patients who had ever smoked.

Supplementary Figures: Kaplan-Meier curves adjusted by sex, age at disease diagnosis and traditional CV risk factors for CV events and cerebrovascular accidents (CVA).

Suppl. Figure A. Cardiovascular events in all RA patients: Anti-CCP+ (solid line) *vs.* Anti-CCP – (dash line).

Suppl. Figure B. Cardiovascular event in anti-CCP positive patients: Allele C (dash line) *vs.* Allele T (solid line).

Suppl. Figure C. CV event in anti-CCP negative patients: Allele C (dash line) *vs.* Allele T (solid line).

Suppl. Figure D. CVA in anti-CCP positive (solid line) *vs.* anti-CCP negative (dash line) in the whole sample.

Suppl. Figure E. CVA in anti-CCP positive patients; allele C: dash line; allele T: solid line.

Suppl. Figure F. CVA in anti-CCP negative patients; allele C: dash line; allele T: solid line.
